# Split hand/foot malformation with long bone deficiency associated with *BHLHA9* gene duplication: a case report and review of literature

**DOI:** 10.1186/s12881-019-0839-2

**Published:** 2019-06-14

**Authors:** Chamara Sampath Paththinige, Nirmala Dushyanthi Sirisena, Fabienne Escande, Sylvie Manouvrier, Florence Petit, Vajira Harshadeva Weerabaddana Dissanayake

**Affiliations:** 1 Human Genetics Unit, Faculty of Medicine, University of Colombo, Kynsey Road, Colombo, 00800 Sri Lanka; 2 Faculty of Medicine and Allied Sciences, Rajarata University of Sri Lanka, Saliyapura, Anuradhapura, 50008 Sri Lanka; 3 Laboratoire de Biochimie et Oncologie Moléculaire, CHU Lille, F-59000 Lille, France; 4 Clinique de Génétique Guy Fontaine, CHU Lille, F-59000 Lille, France

**Keywords:** Ectrodactyly, Split hand/foot malformation, Split hand/foot malformation with long bone deficiency, 17p13.3 duplication, *BHLHA9*

## Abstract

**Background:**

Split hand/foot malformation (SHFM) is a group of congenital skeletal disorders which may occur either as an isolated abnormality or in syndromic forms with extra-limb manifestations. Chromosomal micro-duplication or micro-triplication involving 17p13.3 region has been described as the most common cause of split hand/foot malformation with long bone deficiency (SHFLD) in several different Caucasian and Asian populations. Gene dosage effect of the extra copies of *BHLHA9* gene at this locus has been implicated in the pathogenesis of SHFLD.

**Case presentation:**

The proband was a female child born to non-consanguineous parents. She was referred for genetic evaluation of bilateral asymmetric ectrodactyly involving both hands and right foot along with right tibial hemimelia. The right foot had fixed clubfoot deformity with only 2 toes. The mother had bilateral ectrodactyly involving both hands, but the rest of the upper limbs and both lower limbs were normal. Neither of them had any other congenital malformations or neurodevelopmental abnormalities. Genetic testing for rearrangement of *BHLHA9* gene by quantitative polymerase chain reaction confirmed the duplication of the *BHLHA9* gene in both the proband and the mother.

**Conclusions:**

We report the first Sri Lankan family with genetic diagnosis of *BHLHA9* duplication causing SHFLD. This report along with the previously reported cases corroborate the possible etiopathogenic role of *BHLHA9* gene dosage imbalances in SHFM and SHFLD across different populations.

## Background

Ectrodactyly or split hand/foot malformation (SHFM) is a group of genetic skeletal disorders with variable phenotypes. Complete absence or under-development of the central digits with a cleft in the hands and/or feet is the archetypal phenotype. However, SHFM is clinically heterogeneous, varying from slight shortening of a single central digit to monodactyly in extreme cases. Ectrodactyly may occur as an isolated anomaly affecting only one or more limbs, or in syndromic forms with extra-limb manifestations [1]. To date, single gene pathogenic variants, recurrent genomic duplications and balanced or unbalanced chromosomal rearrangements in several chromosomal loci are reported to be associated with isolated forms of SHFM. Most are inherited in an autosomal dominant pattern. However, incomplete penetrance is a common occurrence in affected families. Several genes in chromosomal loci that are known to regulate embryonic development of the limb buds in animal models are implicated in the genetic etiopathogenesis of the SHFM. One of the most frequently described is the *TP6*3 gene that accounts for about 10% of isolated SHFM [2]. Duplication in the 17p13.3 locus is reported to be associated with SHFM with long bone deficiency (SHFLD), frequently involving the tibia and fibula [3, 4]. The SHFLD critical region in the 17p13.3 locus contains the *BHLHA9* (basic helix-loop-helix [BHLH] family member A9) gene. The orthologous gene has been reported to be expressed in the mesenchyme of the developing limb buds in zebrafish and mouse models, thus the *BHLHA9* gene is proposed to regulate human limb development [5].

The clinical and genetic heterogeneity of SHFM and SHFLD render the precise diagnosis of the condition challenging, and genetic testing to detect the underlying chromosomal or genetic defect is necessary for confirmation. Herein, we report the case of a mother and daughter with SHFLD with *BHLHA9* duplication, which is the first reported Sri Lankan family with a molecular genetic diagnosis of ectrodactyly.

## Case presentation

**Fig. 1:**
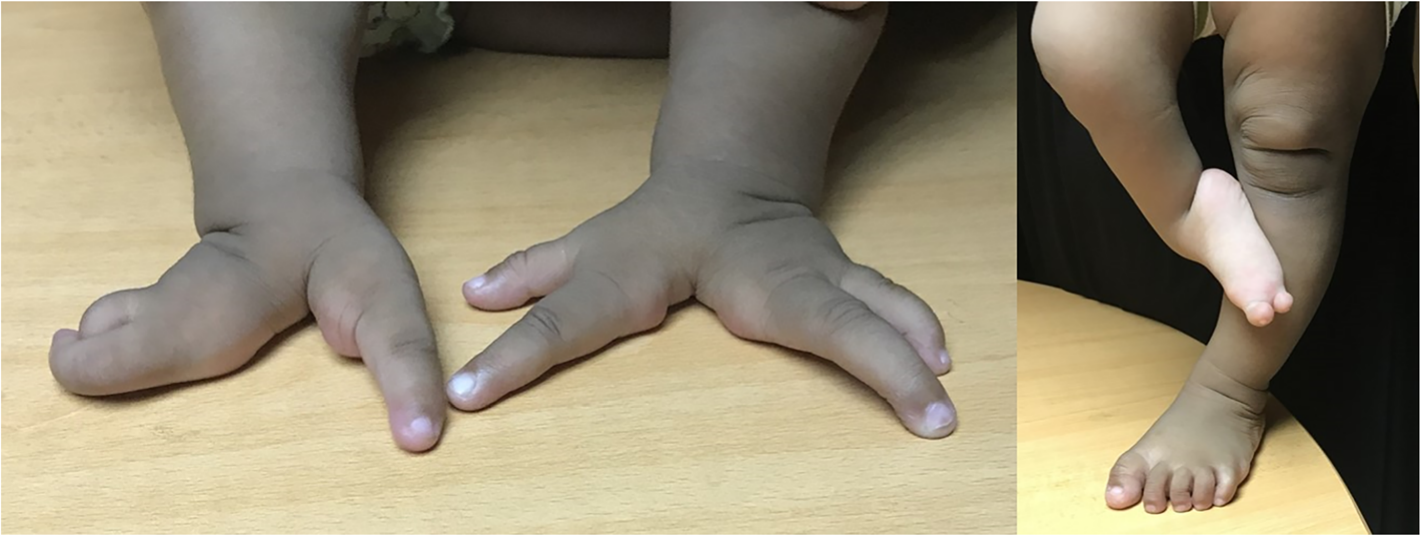
Clinical photographs of the hands and the feet of the proband showing ectrodactyly involving three limbs, with the absence of the third digit on the left hand, absence of 2 digits on the right hand with malformed fingers (except the thumb) and the fixed clubfoot deformity with only 2 toes on the right foot

**Fig. 2:**
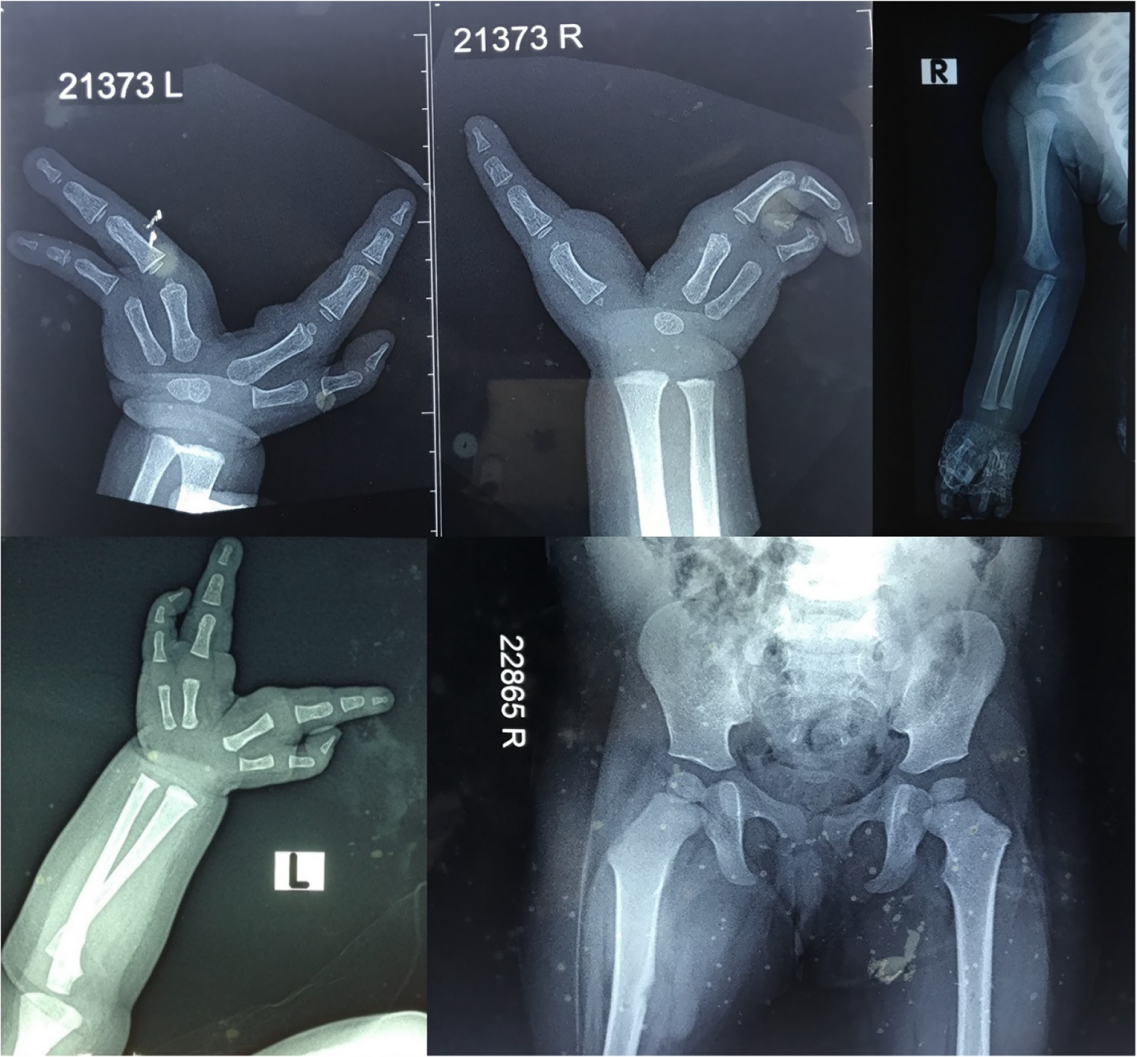
Radiological images of the upper limbs and pelvis of the proband showing complete absence of the metacarpal and the phalanges of the third digit on the left hand and absent metacarpals and phalanges of two digits on the right hand. Rest of the upper limbs and upper femur were radiologically normal

A 4-day-old baby girl was referred from a paediatric tertiary care hospital for the genetic evaluation of bilateral asymmetric ectrodactyly. She is the second child of a non-consanguineous couple; a 25-year-old father and 23-year-old mother. The baby was delivered normally at term following an uncomplicated pregnancy. The birth weight was 2.5 kg and there were no post-natal complications. She had ectrodactyly involving three limbs, with the absence of the third digit on the left hand and the second and third digits on the right hand. The right thumb was clinically normal, but the fourth and fifth digits were malformed. The right foot had fixed clubfoot deformity with only 2 toes (Fig. 1). There was no facial dysmorphism or facial clefts. Radiographs of the upper limbs showed complete absence of the metacarpal bone and the phalanges of the third digit in the left hand and absent metacarpals and phalanges of two digits on the right hand (Fig. 2). During a subsequent evaluation of the proband at the pediatric clinic, right tibial hemimelia was documented in the patient’s medical records by the attending clinician, but the radiological images of the leg were not available for inclusion in this article. Cardiovascular, respiratory and abdominal examination showed no abnormalities. Ultrasonography of the abdomen, brain and bilateral hips were normal.

**Fig. 3:**
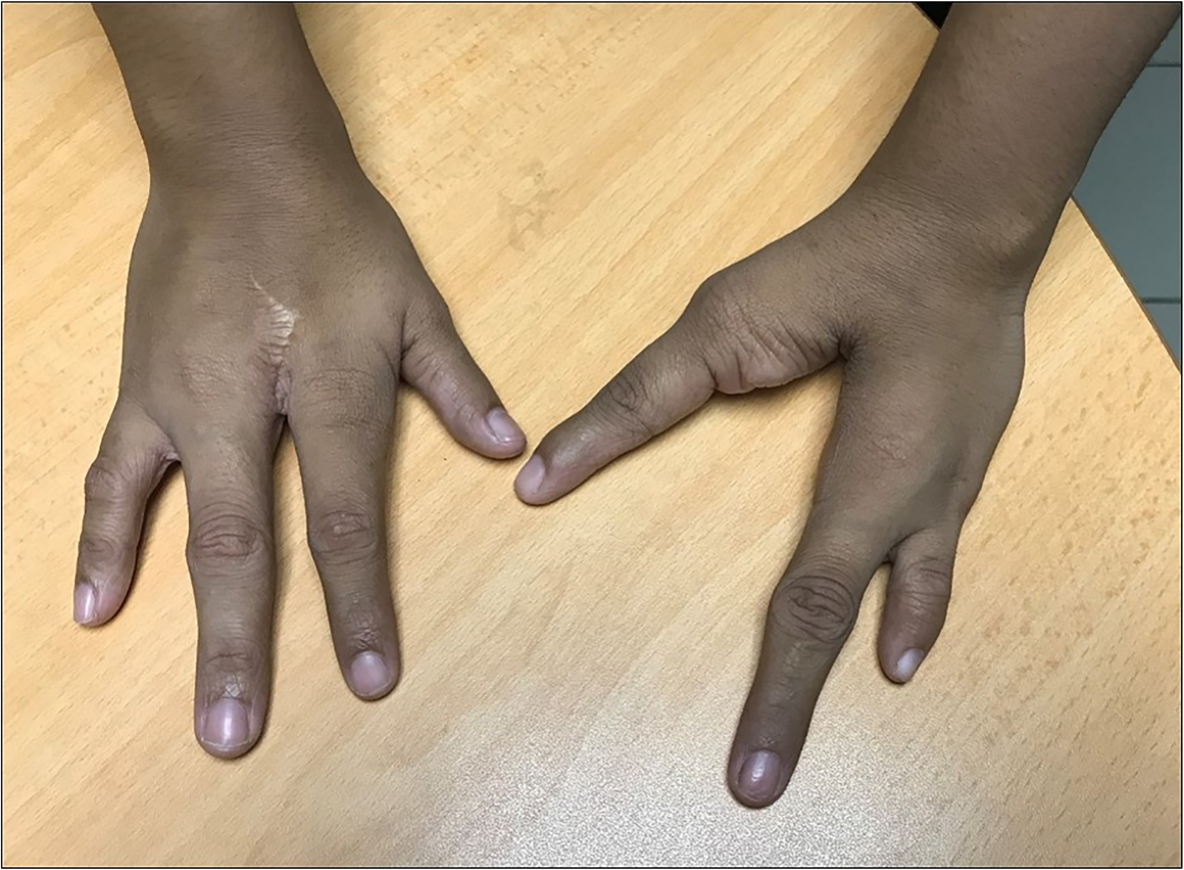
Clinical photographs of the hands of the mother showing bilateral ectrodactyly involving both hands, with the absence of the third digit on the right hand and two digits on the left hand, with bi-phalangeal left 5th digit

The mother also had bilateral ectrodactyly involving both hands, with the absence of the third digit on the right hand and two digits on the left hand. She had bi-phalangeal fifth digit on the left hand (Fig. 3). She had not previously been investigated for this condition and was otherwise healthy without any remarkable events in the medical and obstetrics history. The first child of the couple who was aged 2 ½ years old at the time of consultation had normal growth and development with no congenital anomalies. There were no other family members or close relatives affected with similar limb deformities or other congenital anomalies.

Peripheral venous blood samples were obtained from the baby and the mother with informed written consent. Genomic DNA was extracted from the blood samples and quantitative polymerase chain reaction (qPCR) was performed to identify rearrangements of the *BHLHA9* gene (Chr17:1173858–1,174,565, hg19)(Ref Seq *BHLHA9:*NM_001164405). The *RPPH1* gene (NR_002312) was used as the gene of reference. The qPCR results showed a *BHLHA9/RPPH1* ratio of 1.46 in the baby and 1.50 in the mother confirming the *BHLHA9* gene duplication. The unaffected sibling was not available for genetic assessment of her *BHLHA9* status. Genetic counseling was offered to the family.

At the time of reporting, the baby was 10 months of age. Her body weight was 7.95 kg (25th centile), length was 72 cm (above 50th centile) and head circumference was 44 cm (between 25th and 50th centile). She had age-appropriate developmental milestones. Hearing and visual assessments were normal. Repeat ultrasonography of the brain and the abdomen showed no abnormalities. 2D-echocardiography showed a structurally and functionally normal heart. She is currently being followed up at the pediatric tertiary care hospital and awaiting reconstructive surgery.

## Discussion and conclusions

Ectrodactyly or SHFM is a clinically and genetically heterogeneous group of skeletal disorders with or without extra-limb manifestations. As observed in the present case, the clinical heterogeneity leads to variable expressivity of the malformations among different affected individuals even within the same family [1]. Most of the isolated forms of SHFM; [SHFM1: chromosomal locus 7q21.2, SHFM3: 10q24, SHFM4: 3q28, SHFM5: 2q31] follow the autosomal dominant inheritance pattern. Incomplete penetrance is a common phenomenon in some families. Rare forms of SHFM such as SHFM6 mapped to chromosomal locus 12q13.12 and SHFM2 mapped to chromosomal locus Xq26 have also been described in families that show autosomal recessive and X-linked recessive inheritance patterns, respectively [1, 6].

Duplication at the 17p13.3 locus that was identified in the mother and daughter in the present case is reported to be associated with SHFLD, frequently involving the tibia and rarely other long bones. Some individuals with this duplication are reported to present with isolated SHFM without long bone involvement (Table 1) [3–5, 7–16]. The link between SHFLD and 17p13 duplication was first reported by Lezirovitz et al. in 2008. They identified the chromosomal region 17p13.1–13.3 as the candidate region for SHFLD by linkage analysis and multipoint Lod scores calculations in a Brazilian family with nine affected individuals with SHFM with or without tibial hypoplasia or aplasia [3]. Armour et al., in 2011 investigated 3 families with SHFLD with tibial involvement and narrowed the SHFLD critical region to a 173 kb region at 17p13.3, suggesting *BHLHA9* as a possible candidate gene [4]. The critical region was further narrowed to an 11.8 kb region containing only the *BHLHA9* gene by Klopocki et al. in 2012, after investigating 17 families with 42 individuals affected with SHFLD. They demonstrated that the expression of *BHLHA9* gene in the limb buds of the mouse and zebrafish embryos are restricted to the mesenchyme underlying the apical ectodermal ridge (AER), hence suggesting that BHLHA9 transcription factor regulates the interaction between the AER and the progress zone (PZ) in human limb development and morphogenesis [5].

**Table 1:** Studies describing the 17p13.3 duplication/ triplications associated with SHFM or SHFLD

Reference [No.]	No. of families/ individuals with duplication/ triplication (^a^)	Limb anomalies of affected individuals	Extra-limb manifestations	Special remarks
ezirovitz et al., 2008 [3]	1 family 9 affected individuals	SHFM (4), SHFLD [tibial hemimelia] (3), Tibial hemimelia/aplasia only (2)	No	Autosomal dominant (AD) inheritance/ Variable expressivity (VE)/, Incomplete penetrance (IP), Defined candidate region to 17p13.1 – 17p13.3; ~ 861 kb
Armour et al., 2011 [4]	3 families (?1 with triplication^a^) with 12 affected individuals 7 with duplication: 6 affected, 1 unaffected	Split hand only (3), SHFLD [tibial hypoplasia/aplasia] (9)	No	AD inheritance/ VE/ IP Defined critical region of ~ 173 kb, ?*BHLHA9* or AC016292 duplication
Klopocki et al., 2011 [5]	17 families (out of 56) 82 with duplication: 42 affected, 40 unaffected	SHFLD [tibial hemimelia] (18/31) Only SHFM (5)	No	AD inheritance/ VE/ IP Sex bias: (Male>Female), affected females with more severe phenotype Defined critical region of ~ 11.8 kb encompassing only *BHLHA9* gene
Petit et al., 2013 [7]	2 affected with duplication	Case 1 SHFLD [R/radial agenesis and hypoplastic R/ulna and L/radial hypoplasia] Case 2 SHFM only	Case 1 small ASD	Involvement of radius reported for the first time
Curry et al., 2013 [8]	1 affected 14 families in this report (total of 21 families analyzed) with 17p13.3 duplications that includes *BHLHA9*	SHFLD [tibial hemimelia]	Cleft palate, Mild ID	
Luk et al., 2014 [9]	1 affected fetus^a^ Unaffected mother^a^ both with duplication	Split hands	No	First case with prenatal genetic diagnosis
Petit et al., 2014 [10]	13 families with 42 affected individuals and 19 unaffected obligate carriers; 29 with the duplication (20 affected, 9 unaffected)	SHFLD (18)	No	AD inheritance/ VE/ IP Involvement of radius in 2 individuals Femur hypoplasia in one patient Affected males with more severe phenotype
Al Kaissi et al., 2014 [11]	1 affected Father- bilateral partial syndactyly	SHFM, tibial hemimelia	Sacral hypoplasia, DD, Thrombocyto-penia	
Nagata et al., 2014 [12]	27 families 64 with the duplication/triplication (42/42 affected patients, 22/47 unaffected relatives); 2/1000 Japanese controls positive for duplication	SHFM (29), SHFLD (11), GWC (2)	NR	No sex bias SHFLD and GWC more common in triplications
Nagata et al., 2015 [13]	1 affected child^a^ Unaffected mother^a^ both with triplication	SHFM with tibial aplasia (R)/ hypoplasia (L) and wide R/ distal femoral metaphysis (GWC-like malformation)	No	
Cho et al., 2015 [14]	1 affected fetus with the duplication	SHFLD, campomelia of R/femur, Bilateral agenesis of fibula, bilateral club feet, oligosyndactyly	No	
Fusco et al., 2017 [15]	3 affected with the duplication	SHFLD; tibial hypoplasia (2) One with isolated tibial hypoplasia	One with ASD	
Shen et al., 2018 [16]	1 family with 5 affected individuals, 4 affected fetuses 10 tested (8 individuals, 2 fetuses) 8 with the duplication; 6 affected, 2 unaffected	SFFM (4) SHFLD (1), bilateral femoral hypoplasia	No	
Present report 2018	1 affected child Affected mother	SHFM (?with tibial hemimelia)	No	

*ASD* Atrial septal defect, *DD* Developmental delay, *GWC* Goellop Woolfgang Complex, *ID* Intellectual disability, *R* Right, *L* Left

^a^Individuals with 17p13.3 triplication

According to GnomAD SV database, the population frequency of *BHLHA9* duplication is estimated to be 5 × 10^− 5^ [17]. Chromosome 17p13.3 duplication encompassing the *BHLHA9* gene was reported to be less than 50% penetrant in the OMIM database [18], and Klopocki et al. also reported that only approximately 50% of individuals with the duplication manifest the phenotype [5]. A notable feature is the variable expressivity of the condition even within the same family as observed in the present case [3–5]. In addition, Klopocki et al. observed a sex bias resulting in more affected males than females, with higher risk of unaffected carriers having an affected male offspring compared to an affected female offspring (36% vs. 15%), but more severe phenotypic expression in females when affected [5]. In 2013, Petit et al. reported 2 cases of SHFLD, one with radial agenesis, thus expanding the phenotypic spectrum of SHFLD [7]. The first case of SHFM with a prenatal diagnosis of 17p13.3 triplication was reported in 2014 [9]. In the same year, there were two reports describing the molecular genetic basis of SHFLD in two large cohorts of patients from 13 families in France [10] and 51 families in Japan [12]. They reported the rare but possible involvement of the radius and the femur in patients with SHFLD with *BHLHA9* duplication or triplication [10, 12]. These studies suggest that 17p13.3 duplication is the most common cause of SHFLD. The summary of previous studies describing the 17p13.3 duplication associated with SHFM and SHFLD are presented in Table 1.

The *BHLHA9* gene in the minimal critical region of 17p13.3 in SHFLD encodes a bHLH transcription factor that is observed to regulate the embryonic limb bud development and morphogenesis in animal experiments. The importance of *BHLHA9* gene in the development of limbs in humans was implied by few recent studies that described the association of homozygous missense variants in the *BHLHA9* gene with the mesoaxial synostotic syndactyly with phalangeal reduction [19, 20] and with complex camptosynpolydactyly [21]. In a recent study using *BHLHA9* knockout mice, Kataoka et al. reported that the *BHLHA9* gene is expressed in the early embryonic stages on both the ventral and dorsal surfaces of the PZ. The *BHLHA9* knockout mice showed transient aberrant expression of *TP63* and *FGF8* genes, indicating that the *BHLHA9* gene regulates the expression of *TP63* and downstream genes [22]. A previous study also using *BHLHA9* knockout mice, suggested that the complete deletion of the *BHLHA9* causes syndactyly due to reduced apoptosis between digital rays [23], however Kataoka et al. showed the expression of *BHLHA9* occurs earlier in the embryo than the interdigital cell apoptosis stage [22]. The *BHLHA9* gene is an interesting example of the gene dosage effect; a single gene giving rise to two different phenotypic effects (i.e. syndactyly and ectrodactyly) depending of the gene dosage, however the exact molecular mechanisms of pathogenesis of these conditions still remain unclear.

In the published literature, there are several reports of 17p13.3 duplication involving the *BHLHA9* gene region that presented predominantly with developmental delay, intellectual disabilities, autism and occasional brain malformations. Only a small number of these patients presented with subtle hand foot malformations and the limbs were completely normal in most cases [8, 24–27]. These data along with the non-penetrance observed in SHFLD families suggest a complex genetic etiology for SHFLD with the involvement of additional genetic modifiers. Thus, increased *BHLHA9* gene dosage by itself is not sufficient to produce the phenotypic effect. Moreover, the duplication breakpoints in patients with SHFLD were observed to occur closer to the *BHLHA9* region*,* compared to patients with 17p13.3 duplication without SHFLD, but with other phenotypic manifestations such as neurodevelopmental abnormalities and facial clefts. This suggests that the disruption of adjacent putative regulatory elements (e.g. in the *ABR-TUSC5* region) of the *BHLHA9* gene could be an additional contributory factor in the causation of SHFLD [8, 27, 28].

The clinical and genetic heterogeneity of SHFM/SHFLD render the precise diagnosis of this condition challenging, and genetic testing is therefore essential for confirmation. Sowińska-Seidler et al. have proposed a genetic diagnostic flow-chart that would be useful in genetic diagnosis of SHFM. Considering the high frequency of chromosomal rearrangements and genomic micro-duplications among patients with SHFM, genome-wide array comparative genomic hybridization (array CGH) is often considered as the first line of genetic testing. Alternatively, targeted qPCR or multiplex ligation-dependent probe amplification (MLPA) could be used to detect copy number variations associated with SHFM3 and SHFLD. These were shown to have a diagnostic yield of 30–46% [6]. Even with the precise genetic diagnosis, which in itself is a challenge, providing genetic counseling for families with SHFM is a bigger challenging task, due to the reduced penetrance, variable expressivity and the sex bias, which make the risk assessment and the phenotypic prediction a rather daunting task.

## Data Availability

All data generated in this study are included in this published article.

## Abbreviations

AER: Apical ectodermal ridge
*BHLHA9*: Basic helix-loop-helix [BHLH] family member A9
CGH: Comparative genomic hybridization
MLPA: Multiplex ligation-dependent probe amplification
PZ: Progress zone
qPCR: Quantitative polymerase chain reaction
SHFLD: Split hand/foot malformation with long bone deficiency
SHFM: Split hand/foot malformation
